# Effects of Reallocating Time Spent Engaging in Sedentary Behavior and Physical Activity on Mortality in Older Adults: ELSIA Study

**DOI:** 10.3390/ijerph18084336

**Published:** 2021-04-19

**Authors:** Lucas Lima Galvão, Rizia Rocha Silva, Renato Mendonça Ribeiro, Sheilla Tribess, Douglas de Assis Teles Santos, Jair Sindra Virtuoso Júnior

**Affiliations:** 1 Research Center on Physical Activity, Health and Aging, Department of Sport Sciences, Institute of Health Sciences, Federal University of Triangulo Mineiro, Uberaba, Minas Gerais 38025-180, Brazil; lucasgalvao07@gmail.com (L.L.G.); riziarochasilva@gmail.com (R.R.S.); renatomribeiro@uberabadigital.com.br (R.M.R.); sheillatribess@yahoo.com.br (S.T.); 2 Department of Education, University do Estado da Bahia, Teixeira de Freitas, Bahia 45992-255, Brazil; datsantos@uneb.br

**Keywords:** physical activity, sedentary, health, behavior change

## Abstract

Background: The objective of the study is to investigate the effects of reallocating time spent engaging in sedentary behavior (SB) and physical activity on the risk of mortality. Methods: In all, 332 older adult low-income and low-education populations participated in the study. At the end of the study, 273 of the participants were alive and 59 had died. Time spent undertaking moderate to vigorous physical activity (MVPA) and SB was assessed using the international physical activity questionnaire. The Cox proportional hazards regression model was used. Results: The replacement of time spent engaging in SB with MVPA reduced the risk of mortality from all causes in the older adults, resulting in reductions in mortality risk of between 10% and 46%. Conclusion: A reduction in the risk of mortality in older adults was observed when time spent in SB was replaced with the same amount of time in MVPA for all times tested.

## 1. Introduction

The benefits of regular physical activity (PA) are well described in the literature, with exercise being associated with reductions in the risk of cardiovascular disease [1,2], type 2 diabetes mellitus [3] and various types of cancer [4], increased or maintained bone mineral strength and density [5], and a reduced risk of mortality [1,6]. PA is defined as any activity performed by skeletal muscles with energy expenditure [7]. Conversely, sedentary behavior (SB) is associated with several health risks [8], defined as activities performed in a sitting or reclining position with energy expenditure ≤ 1.5 METs (metabolic equivalent of task) [9], also classified as “sitting time” in some studies [10].

Due to advancing age, the time spent in PA tends to fall, and SB is more frequent. For this reason, the health problems of elderly populations tend to increase. The regular practice of PA associated with a reduction in SB, results in promoting an improvement in life expectancy [8]. Despite these benefits, Physical Activity (PA) levels have decreased considerably because of technology developments that has turned it into a public health problem [11]. Therefore, the majority of elderly people tend not to follow the minimum PA recommendations [12].

Considering the number of hours in a finite day, SB and PA are interdependent, meaning that increasing the time spent engaging in one of these behaviors decreases the time spent in the other [13]. The benefits of reduced SB time depend not only on the reduction in time itself, but also on the type of activity to which this time is reallocated [14]. These effects can be investigated by isotemporal substitution modeling, which is commonly used in the field of PA epidemiology [10,14,15]. This model offers specific insights into the classes of association of different times and behaviors [16] with different outcomes, including mortality [10,14,15]. Isotemporal substitution modeling not only controls for the effect between activities (PA vs. SB), but also captures the effect of time substitution and reduces heterogeneity in associations, assisting in public health recommendations [16,17].

Few research efforts have investigated the associations between substitutions of PA and SB with mortality outcomes in longitudinal studies. These studies have been carried out in different countries, such as the National Health and Nutrition Examination Survey (NHANES) [18,19,20], The collaborative study between National Institutes of Health (NIH) and AARP (formerly known as the American Association of Retired Persons) (NIH-AARP) Diet and Health Study Diet and Health Study [21], Reasons for Geographic and Racial Differences in Stroke (REGARDS) [22] and The Cancer Prevention Study—II Nutrition Cohort (CPS-II NC) [23], all of which were carried out in the United States of America, the UK Biobank study in the United Kingdom [24], the 45 and Up Study in Australia [10,15] and the Sweden Attitude Behavior and Change (ABC) in Sweden [14,25].

No studies, at least as far as we know, have focused on the Brazilian older adult population, specifically, older adults within the low-income and low-education population. This population is made up of 473 subjects. The majority of these individuals are female (*n* = 285) aged between 60 to 69 years old (*n* = 254) [26], whose values of sedentary time vary according to age group. For men and women, SB ranged from 189.2 and 210.2 min/day, to 648.5 and 606.2 min/day in the 60–69 years old age group; 222.0 and 283.5, and 246.6 min/day to 660.5 and 680.0 min/day in the 70–79 years old age group; 280.0 and 204.3 min/day to 671.4 and 778, 8 min/day for the 80 years old or more age group [27], with a prevalence of frailty of 22.1% [28].

This information has both clinical and public health relevance and is valuable for the development of more precise and specific recommendations, as well as recommendations and interventions aimed at practicing physical activity and reducing sedentary behavior. It is also of great importance when considering the substitution relationships of one behavior for another. This can be used to elucidate which health benefits this exchange will promote, which in this specific case, is the relationship of PA and SB with mortality. Thus, the aim of the study was to estimate the hypothetical effects of reallocating SB and PA time on mortality in older adults.

## 2. Materials and Methods

### 2.1. Study Design

This research is part of the *Estudo Longitudinal de Saúde do Idoso de Alcobaça*–Bahia, Brazil (ELSIA), a prospective population-based cohort study using an exploratory survey method.

### 2.2. Participants

Baseline data collection from July to September 2015 was carried out by interviewers previously trained in a pilot study, addressing the instruments and reducing possible data collection errors. The elderly population of the municipality consisted of 2047 people aged ≥ 60 years old, from which 1024 represented the total of elderly residents in the urban area of the municipality [29]. The initial sample of the present study consisted of 743 elderly people registered in the Family Health Strategy. During data collection, 54 elderly people refused to participate, 58 individuals were excluded because they did not meet the final sample of the baseline. The final sample consisted of 473 older adults of both genders aged ≥ 60 years old, registered in the Family Health Strategy. Expansion, qualification, and consolidation of primary care was conducted in order to favor reorientation of the work process with greater potential to deepen the principles. Guidelines and fundamentals of primary care were assessed in order to expand the resolution and impact on the health situation of people and communities, and in order to provide important insights into cost-effectiveness. During the baseline, *n* = 270 people were lost, with 54 refusals, and 58 were excluded because they did not meet the inclusion criteria, and 158 elderly people were not found after three attempts with a final sample of 473 elderly men and women. As regards home visits, the researchers used the data provided by the Municipal Health Department of Alcobaça as a reference. Contact was made with elderly individuals through home visits. We informed them about the objectives of this study and requested their participation in the research voluntarily. After their acceptance, a free and informed consent form (ICF) was delivered, and submitted to a questionnaire that was applied as an interview.

The exclusion criteria were as follows: severe cognitive impairment according to the Mini Mental State Examination (MMSE), adapted for the Brazilian population [30]; severe difficulty in visual and/or hearing acuity; wheelchair use; severe sequelae of stroke (stroke) with localized loss of strength; terminal illness.

Of the participants included in the first wave of the study, 105 were not located and 36 moved to another city, totaling 141 segment losses. Of the 332 returns, 59 participants died and 273 were alive. The follow-up took place from January to February 2020. Details of the participants are provided in the Supplementary Materials.

### 2.3. Ethical Procedures

The study protocol and procedures were carried out in accordance with the Declaration of Helsinki and were previously approved by the Human Research Ethics Committee of the Human Research Ethics Committee of the Federal University of Triângulo Mineiro (number 966.983/2015) and the State University of Bahia (number 3.471.114/2020).

### 2.4. Mortality

The vital status was determined through telephone follow-up, information from family members with presentation of the death certificate, information obtained from the municipal registry and/or consultation on the website of the Court of Justice of the State of Bahia. For this study, mortality from all causes was included, and the follow-up time from the beginning of the survey to death, censor or the end of the second wave (29 February 2020) was calculated.

### 2.5. Physical Activity and Sedentary Behavior

The level of PA and the time exposed to SB were assessed using the International Physical Activity Questionnaire (IPAQ), validated for older Brazilian adults [31,32]. The level of PA was determined from activities of moderate to vigorous intensity (MVPA) performed for at least 10 continuous minutes, assessed in the PA domains of leisure, work, transportation and home. For characterization, the population was dichotomized as sufficiently active (≥150 min/day of moderate intensity physical activity, 75 min/day of vigorous intensity physical activity or a combination of both) or insufficiently active [33]. Sedentary behavior was determined by the time spent sitting, assessed from the questions of sitting time on a typical day of the week (“How much time do you spend sitting during a weekday?”), and a regular weekend day (“How much time do you spend sitting on a weekend day?”). We determined the total sitting time, measured as the number of minutes per day from the weighted average of sitting time on a weekday and weekend day: (sitting time on a weekday × 5 + sitting time on a day weekend × 2/7). The SB time was considered elevated from the 75th percentile (P75 = 540 min/day).

### 2.6. Sleep Assessment

The measurement of nocturnal sleep time was performed using a specific question from the Pittsburg Sleep Quality Index [34], validated for Brazilians (PSQI-BR) [35]: “during the past month, how many hours of sleep did you have per night?”. This measure was used to calculate the total time for activities during the day.

### 2.7. Potential Confounding Factors

There were several potential confounding factors in this study: gender (male and female); age; income; race/ethnicity (white, black, brown) according to the evaluator’s perception; marital status (with a partner or without a partner); number of hospitalizations in the past 12 months; number of medicines, only considering medicines prescribed by a doctor; number of diseases, counted from a list of diseases where the respondent answered whether or not they had the disease; body mass index (BMI), calculated from weight and height measured using a digital scale with a portable stadiometer (Wiso W721); use of tobacco (yes or no), self-reported by the subject.

### 2.8. Data Analysis

Data were entered in duplicate in Epidata software (version 3.1b) (EpiData, Odense, Denmark), and all statistical analyses were performed using SPSS software (version 23.0) (IBM, Armonk, NY, USA). To test the normality of the data, the Komolgorov–Smirnov test was used. Descriptive statistics with dispersion calculations and absolute and relative frequencies were used to characterize the sample. The chi-square (qualitative variables) and Mann–Whitney U tests (quantitative variables) were used to compare vital status and descriptive variables.

The isotemporal substitution model was used to investigate the theoretical consequences of replacing one behavior with another for the same amount of time. This method allowed us to take into account the interdependence of activities of different intensities, making more realistic assumptions that an increase in one behavior will be accompanied by a decrease in duration equal to others, while the total time in all behaviors is kept constant [16,17].

To verify the hypothetical effects of replacing MVPA and SB on mortality risk, Cox proportional hazard regression models were used together with hazard ratio (HR ratio estimates and 95% confidence intervals (CI) for all-cause mortality, with survival time in months. This technique is analogous to multiple logistic regression and allows us to estimate the hazard ratio of a primary exposure variables while controlling for the effects of other covariates. An important assumption of this method is that the hazard ratio of the primary exposure (and also of any other covariates in the model) remains constant over time. This assumption can be tested by introducing a cross-product term of a specific variable of interest (x) by time (t) into the model and testing for statistical significance [36]. The models were adjusted for sex, age, income, number of hospitalizations, number of medications, race/ethnicity, BMI, number of diseases and smoking. The replacement times tested were 10, 20, 30, 40, 50 and 60 min of the MVPA variables and the SB time, while the sleep time remained constant in the model. Before the isotemporal analysis, all the assumptions of the proportional risks were tested, with no violations observed. A significance level of 5% was adopted for all analyses.

## 3. Results

The analysis included a sample of 332 individuals. Table 1 presents the sociodemographic, health and behavioral characteristics of participants according to their vital status. On average, people who died were insufficiently active, engaged in SB for a longer amount of time, were older, required more medications and had a lower mean BMI than those with living vital status.

Table 2 shows the median, standard error and interquartile range of the variables MVPA, sleep and SB included in the isotemporal replacement model.

The isotemporal replacement model is shown in Table 3. In all the tested models of 10, 20, 30, 40, 50 and 60 min, replacement of MVPA time with time spent engaging in SB proved to be a risk factor for mortality. Conversely, replacing the SB time was shown to be a protective factor, with reductions in mortality risk of 10% to 46% for replacement of 10 and 60 min, respectively. The amount of time spent sleeping was not included in the behavior reallocation analysis and was kept constant in the model.

## 4. Discussion

This study aimed to estimate the hypothetical effects of substitution of SB and MVPA time on mortality in older adults. The substitution of time spent engaging in SB with MVPA reduced the risk of mortality from all causes in our older adult population. These associations were proportional to the amount of time reallocated for each activity, with reductions in the risk of mortality ranging from 10% to 46%. Conversely, by replacing the discriminatory time in MVPA for time in SB, the mortality risk was increased by 10% to 82% for 10 and 60 min, respectively. Additionally, among the older adults who died, a higher number of diseases, lower BMI, and increased consumption of medication was reported.

The advance of age among the elderly is related to greater expenditure on health products, especially in the exacerbated use of medications [37]. For a population with low economic status and low levels of schooling, this aspect of senescence added to the interrelations of secondary aging enhancing worsening health conditions and family suffering. 

The indication that small inversions of sedentary behavior for moderate and vigorous physical activities may reduce early mortality rates is more impactful for this population, given the financial conditions and low access to health services.

Age tends to be the main risk factor for mortality among older individuals (the older an individual is, the greater chance of death due to physiological and morphological changes). In addition, elderly people also tend to live with a greater number of diseases referred to multimorbidities [38,39,40]. Additionally, an increase in such diseases has been observed due to the consumption of medicines associated with aging [37]. These factors may contribute to the growing risk of death. In this way, the regular practice of physical activity may contribute to reduce this risk of death and may improve survival [6]. In majority those who died during follow-up will have shown lower BMIs, which corroborates other studies found in the literature. Namely, it was observed in some meta-analyses, that a higher risk of mortality is associated with BMI in individuals with a BMI < 23.0 [41].

The main advantage of the isotemporal model is that it allows for the comparison of the substitution of time spent engaged in one behavior with the same time in another behavior, which can be useful for producing recommendations on how to use discretionary time in a way that is beneficial to health [14]. Reductions in mortality risk associated with regular PA practice at different times and intensities have been evidenced by several studies [6,10,15,42,43,44,45]. A population-based study in Sweden assessed the theoretical consequences of replacing sedentary time with the same duration of moderate and vigorous PA on mortality in 851 individuals aged 50 to 89 years, and found a 24% reduction in mortality risk when sitting time was replaced with moderate intensity PA for 30 min/day. However, no beneficial associations were found for vigorous PA [14].

A longitudinal study performed in Australia showed a 12% reduction in the risk of mortality when sitting time was replaced with moderate PA for 60 min/day [15]. In contrast to the Swedish study, there was a 31% reduction in the risk of mortality from all causes when SB was replaced with vigorous PA [14]. This demonstrates that PA can reduce mortality rates when performed at different intensities, depending on other characteristics of the population.

Other studies have also found significant reductions in the risk of mortality from all causes by replacing the SB time with mild-intensity PA for 30 min/day, with values of 4% [24], 11% [14], 14% [20] and 17% [22]. Furthermore, decreasing the time spent engaging in SB by 60 min/day was reported to reduce the risk by up to 18% [19], indicating that when it is not possible to perform MVPA, activities of light intensity are also beneficial to health.

In several studies, SB has been found to cause harm to the health of populations in general, including older adults [42,43]. In our study, when MVPA time was replaced with time engaged in SB, an increase in mortality risk was observed, with the magnitude of the association dependent on the amount of reallocated time. An Australian study, using the same isotemporal approach, evaluated sleep times in replacement models for 201,129 middle-aged and older adults who slept for ≤7 h/day or >7 h/day and found reductions in the risk of mortality when replacing sitting time with standing, walking and practicing MVPA, with reductions of 5%, 14% and 12%, respectively [10].

Although PA and SB are commonly studied in isolation, so that an increase in one in relation to the other causes different responses in mortality rate, they should be considered together as they present strong synergy [46] to the point of being antagonists.

These findings may be important in the preparation of recommendations for PA and SB for elderly populations. However, it is necessary to be cautious with the interpretation and implementation of specific measures, since other public measures implemented in Brazil have not shown positive results for the increase in PA practice; consequently, not demonstrating its health potential [47]. The number of people who have been engaging in leisure-time physical activity in Brazil has grown annually since 2006, with a greater increase among women and younger adults. Consequently, this behavior increases the percentage of physically active individuals. However, differences in relation to age groups and level of education in contexts where a low increase in PA practice is observed among elderly people aged 65 and over, and in the same sense in people with low education, people with higher education have presented a higher prevalence of PA practice in relation to those with low education. Such increases in PA on leisure between 2009 and 2016 of corresponds 0.79pp/year in the range of 0–8 years of study, from 0.86pp/in the range of 9–11 years of study, and in the range over 12 years of study an increase of 1.26pp/year [48]. 

As limitations of our study, we highlight the use of self-reported measures, which can cause forgetfulness or evaluation bias. However, the evaluators were properly trained in order to reduce the occurrence of bias, and they did not allow the evaluation of PA classified as light intensity, so it could not be included in the isotemporal analysis. Behavior changes over the 5-year follow-up period were not evaluated, an aspect that makes it impossible to say whether changes in behavior over time may have influenced the observed associations. Despite the advantages of using the isotemporal model, modeling estimates are based on statistical modeling and not on real behavioral changes. As strengths, we highlight the longitudinal design of the study, which allowed us to determine causalities in a representative sample of the studied population. Additionally, that is the first study to investigate the effects of behavioral substitutions on mortality risk in elderly Brazilians through the analysis of isotemporal substitution.

## 5. Conclusions

The time spent engaging in physical activity proved to be a protective factor against mortality in older adults. The greater the time spent performing moderate to vigorous physical activity, the lower the risk of mortality. Conversely, the greater the time spent engaging in sedentary behavior, the greater the risk of mortality. The isotemporal substitution model indicates that small changes, such as a 10 min transfer of time engaged in SB to physical activity of moderate to vigorous intensity, are capable of reducing the risk of death by up to 10%.

## Figures and Tables

**Table 1:** Sociodemographic, health and behavioral factors according to vital status.

Variables	All	Vital Status		
		Alive	Deceased	*p*
	*n* (%)	*n* (%)	*n* (%)	
Sex				0.655
Male	121 (36.4)	98 (81.0)	23 (19.0)	
Female	211 (63.6)	175 (82.9)	36 (17.1)	
Race/Ethnicity				0.107
White	103 (31.0)	83 (80.6)	20 (19.4)	
Black	126 (38.0)	102 (81.0)	24 (19.0)	
Brown	102 (30.7)	88 (86.3)	14 (13.7)	
Marital Status				0.871
Lives alone	172 (51.8)	142 (82.6)	30 (17.4)	
Accompanied	160 (48.2)	131 (81.9)	29 (18.1)	
Smoking				0.269
No	295 (88.9)	245 (83.1)	50 (16.9)	
Yes	37 (11.1)	28 (75.7)	09 (24.3)	
Physical activity				
≥150 min/day	173 (52.1)	157 (90.8)	16 (9.2)	<0.001
<150 min/day	159 (47.9)	116 (73.0)	43 (27.0)	
Sedentary Behavior				
<540 min/day	168 (50.6)	146 (86.9)	22 (13.1)0.024	
≥540 min/day	164 (49.4)	127 (77.4)	37 (22.6)	
		Median (SE)	Median (SE)	*p*
Age	-	68.00 (0.46)	78.00 (1.28)	<0.001
Income (R$)	-	1576.00 (212.89)	1576.00 (261.65)	0.878
Number of diseases	-	3.00 (0.16)	3.00 (0.40)	0.025
Body mass index	-	26.99 (0.32)	25.29 (0.71)	0.021
Number of Hospitalizations	-	0.00 (0.42)	0.00 (0.15)	0.086
Number of Medicines	-	2.00 (0.13)	3.00 (0.33)	0.001

SE = Standard Error. Age = in years; income = in reais (R$); body mass index = kg/m^2^.

**Table 2:** Average time throughout the day in the activities included in the substitution model.

	Median	Standard Error	IQR
MVPA (min/day)	21.78	4.01	68.57
Sleep (min/day)	437.00	5.89	150.00
SB (min/day)	419.00	9.08	226.07

MVPA: Moderate to vigorous physical activity; SB: sedentary behavior; IQR: interquartile range.

**Table 3:** Isotemporal substitution model of the association of time reallocation in sedentary behavior and moderate to vigorous physical activity in the risk of mortality in the older adults.

Substitution Models	Mortality	
	HR (CI 95%)	HR (CI 95%)
	MVPA (min/day)	SB (min/day)
10 min		
Replacement of MVPA	-	1.10 (1.01–1.20) *
Replacement of SB	0.90 (0.83–0.98) *	-
20 min		
Replacement of MVPA	-	1.22 (1.03–1.44) *
Replacement of SB	0.81 (0.69–0.96) *	-
30 min		
Replacement of MVPA	-	1.35 (1.05–1.72) *
Replacement of SB	0.73 (0.57–0.94) *	-
40 min		
Replacement of MVPA	-	1.49 (1.07–2.07) *
Replacement of SB	0.66 (0.48–0.92) *	-
50 min		
Replacement of MVPA	−0.60 (0.40–0.90) *	1.65 (1.10–2.49) *
Replacement of SB		-
60 min		
Replacement of MVPA	-	1.82 (1.12–2.98) *
Replacement of SB	0.54 (0.33–0.89) *	-

CI: confidence interval; HR: hazard ratio; MVPA: moderate to vigorous physical activity; SB: sedentary behavior. HR adjusted for sex, age, number of medications, income, number of hospitalizations, race/ethnicity, marital status, body mass index, number of diseases and smoking status. * *p* < 0.05.

## Supplementary Materials

The following are available online at https://www.mdpi.com/article/10.3390/ijerph18084336/s1, Figure S1: Participants included in the study.
