# Supplementary figures and images for: Effects of Reallocating Time Spent Engaging in Sedentary Behavior and Physical Activity on Mortality in Older Adults: ELSIA Study
Source: Int J Environ Res Public Health. 2021 Apr 19;18(8):4336. doi: 10.3390/ijerph18084336 (PMC8074045; doi:10.3390/ijerph18084336)

BASELINE

FOLLOW-UP

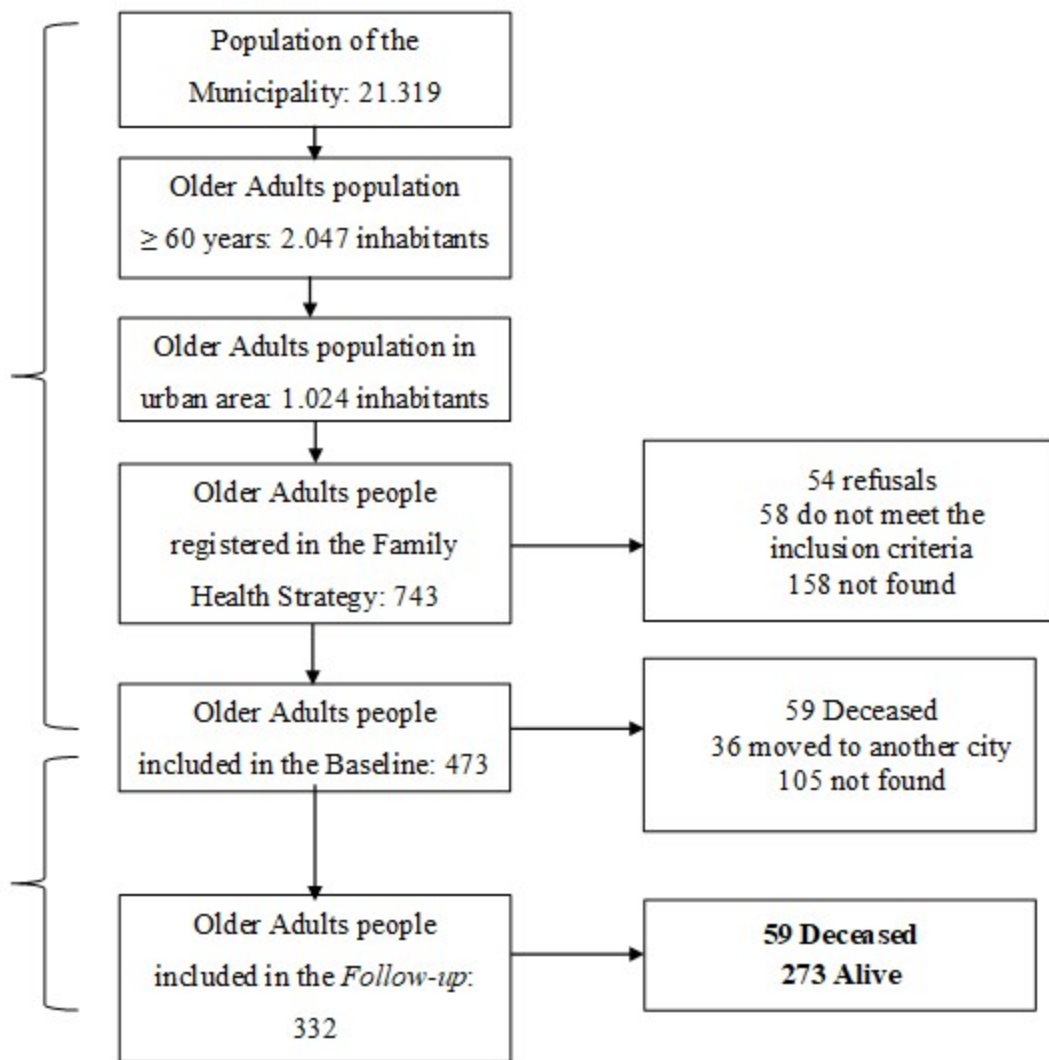

Supplement: Supplementary file 1 [file ijerph-18-04336-s001.zip › ijerph-1157545-supplementary.pdf]
